# Dental disorders in sows from Swedish commercial herds

**DOI:** 10.1186/s13028-020-00521-7

**Published:** 2020-06-04

**Authors:** Anna Malmsten, Nils Lundeheim, Anne-Marie Dalin

**Affiliations:** 1 Department of Clinical Sciences, Swedish University of Agricultural Sciences, PO Box 7054, 750 07 Uppsala, Sweden; 2 Department of Animal Breeding and Genetics, Swedish University of Agricultural Sciences, PO Box 7023, 750 07 Uppsala, Sweden

**Keywords:** Dental disease, Sow, Sweden

## Abstract

Knowledge on dental disorders in commercial sows is limited although such conditions may have important animal welfare implications. In a pilot study, the dental and periodontal health of 58 sows (Landrace*Yorkshire-crosses) from 8 Swedish commercial pig herds, slaughtered at one abattoir, were investigated. The oral cavity was inspected and abnormalities were recorded on a dental chart modified for pigs. Dental abnormalities, absence of teeth, supernumerary teeth, tooth fractures, signs of caries, and malalignment were recorded. The study revealed that 19% of the sows had supernumerary teeth and 59% of the sows missed at least one tooth. Periodontitis, calculus and malalignment were observed in 33%, 45% and 17%, respectively. Tooth wear was very common both in incisors (total 83%) and in premolars/molars (total 84%). One or more tooth fractures (between 1 and 6 per sow) was found in 41%. Signs of caries was found in 9%. In order to assess oral health, three indices were used: calculus index (CI), periodontal index (PDI) and tooth wear index (TWI). Severe periodontitis, tooth wear in incisors and tooth wear in premolars/molars were found in 7%, 34% and 35%, respectively. With respect to animal welfare, the etiology and the effects of the disorders on health, stress and pain need to be investigated.

## Findings

A Swedish study on wild boars showed that a high proportion of supplementary fed animals suffered from dental lesions [1]. For commercial pig herds, attention has been given to problems in piglets after teeth clipping [2], but there has been less focus on dental health issue in adult animals. Few studies on the tooth health of sows in commercial herds have been published [3–5]. In humans, it is well known that periodontal infections may lead to coronary heart disease [6], artery endothelial dysfunction and systemic inflammation [7] but whether this is the case in pigs is to our knowledge not known. In this study, the dental and periodontal health of sows (Landrace*Yorkshire-crosses) from 8 Swedish commercial pig herds was investigated. The heads (n = 58) were collected at one abattoir at ordinary slaughter (permit no SE3801001912, Swedish Board of Agriculture). It was not possible to get detailed information about all individual sows due to loss of ear marks so individual background data were excluded from the study. According to data from five herds, age varied between four and 7 years (n = 35, mean 6.1 ± 1.3 SD). To enable examination of the oral cavity, the jaws were opened by lateral incision through the masseter muscle and manually separated. The oral cavity was inspected and abnormalities were recorded on a dental chart modified for pigs (Additional file 1) [1]. All examinations were made by the same observer (AM). Dental abnormalities, absence of teeth, supernumerary teeth, tooth fractures, caries, and malalignments were recorded. In order to assess oral health, three indices were used: calculus index (CI, 0, 1–3), tooth wear (TW, 0, 1–3), and periodontal index (PDI, 1-3), (Additional file 2) [1]. The severity of the lesion increased with index number. Spearman rank correlations between numerically scored teeth findings were calculated using the SAS software (SAS Inst. Inc., Cary, NC, USA).

**Fig. 1:**
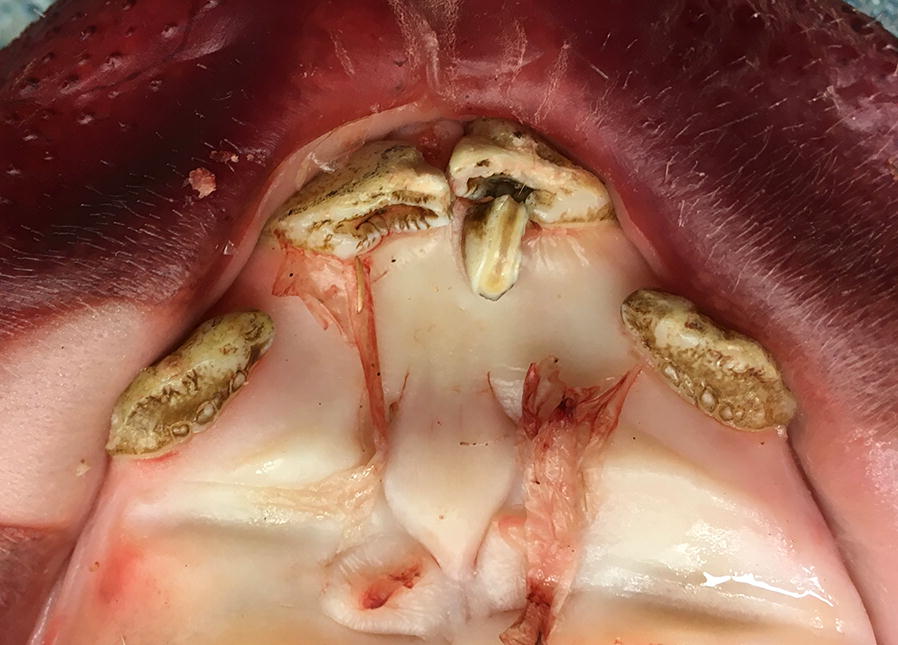
Maxilla of a sow, one supernumerary and inclined tooth (incisor)

**Fig. 2:**
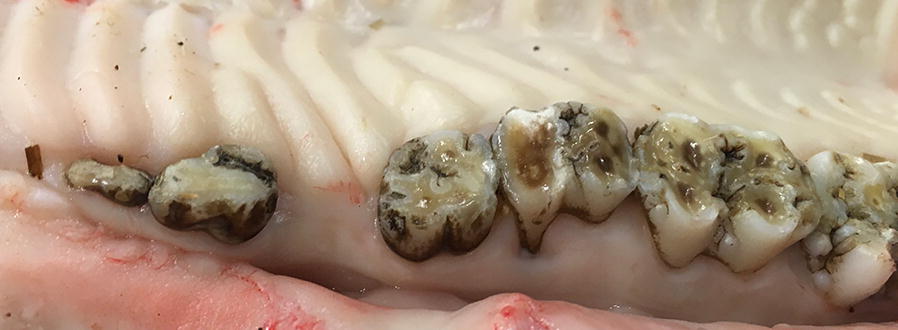
Absence of third premolar and severe periodontitis on second molar, the root is uncovered

The study showed that 19% of the sows (n = 11) had supernumerary teeth (Fig. 1) and that 59% (n = 34) missed at least one tooth. About 50% of the missing teeth were premolars (Fig. 2). The cause of tooth missing could not be assessed. By macroscopic observation, differentiation between hypodontia (congenital absence of one or more teeth), failure to erupt, and tooth loss for other reasons cannot be made.

Calculus was found in 45% of the sows (CI 1 = 3%, Cl 2 = 16%; CI 3 = 26%) while periodontitis was found in 33% of the sows (PDI 1 = 7%; PDI2 19% and PDI 3 = 7%). In the sows with PDI3, defined as gingival recession exposing > 70% of the root, the teeth were loose. Dental malalignment was found in 28% of the sows. Tooth wear was also very common and observed in incisors (83%) as well as in premolars/molars (84%). Severe tooth wear was found in both incisors (34%) and molars (35%). One or more tooth fractures (between 1 and 6 per sow) was detected in 41% (n = 24). Fractures were more common in incisors and found more often in the mandible than in the maxilla. The most severe fractures were observed in incisors but also a few cases were found in premolar/molars. Caries was found in 9%. There was a negative correlation between fracture and tooth wear (p < 0.001) and positive correlations between tooth wear incisors and tooth wear premolar/molars (p < 0.001), between periodontitis and tooth wear (p < 0.05) as well as between calculus and tooth wear (p < 0.05).

The results show that dental disorders are common among Swedish commercial sows and different from those found in female wild boars in Sweden [1]. The domestic sows from commercial pig herds had some disorders that may be of genetic origin (e.g. supernumerary teeth, absence of teeth, malalignment) and which were uncommon in wild boars. All these three disorders may lead to abnormal wear and also predispose to dental diseases such as caries and periodontitis, e.g. due to impaction of food between teeth. A genetic basis for certain anomalies of the teeth is well known in humans [8].

In the present study, high proportions of tooth wear were found both in incisors and premolars/molars. One possible explanation could be that the sows had been bar-biting, which may be a behaviour around feeding [9]. High frequency of tooth wear (71%) was also reported from a recent Finnish study on commercial sows found dead or being euthanized [5]. According to Davies et al. [10], tooth wear was found in both outdoor (28%) and indoor sows (30%). In wild boars, tooth wear was more common in molars than in incisors [1], which may be explained by the wild boars rooting behaviour resulting in mastication of soils and gravels. Fractures were observed more on incisors than on premolars/molars, which also may be due to the sow behaviour to chew on stable interior.

Ala-Kurikka et al. [5] classified the dental disorders in ‘tooth wear, fracture, periodontal disease and calculus’ and showed that fractures were the second most common dental disease. The proportion of periodontitis was higher in the present study (33%) than in the Finnish study (26%) [5]. The reason may be different assessment of tooth disorders but also the type of sows examined. In the present study the sows were sent to an abattoir, i.e. the sows were considered fit for transport and human consumption. In spite of this, many of the slaughtered sows (26%) had severe periodontitis (PI2 and PI3).

The present study, which was based on a limited sample size, clearly showed that tooth disorders are common in at least some Swedish commercial sow herds. More studies on adult pigs are needed to determine the effect of tooth disorders on sow welfare and health and the association between dental health and culling as well as the effect of housing and feeding regimes.

## Supplementary information

**Additional file 1:** Dental examination chart. Description: Incisors (I), canines (C), premolars (P) and molars (M) are numbered in accordance with their appearance in primitive eutherian dentition (lower-case letter indicates deciduous teeth).

**Additional file 2: Table S1.** Indices used to evaluate dental health in Swedish commercial sows [1]

## Data Availability

Data about the result is available from the corresponding author upon reasonable request.
