# Supplementary material for: Dental disorders in sows from Swedish commercial herds
Source: Acta Vet Scand. 2020 Jun 4;62:27. doi: 10.1186/s13028-020-00521-7 (PMC7273662; doi:10.1186/s13028-020-00521-7)
Supplement: Supplementary file 1 — Additional file 1: Dental examination chart. Description: Incisors (I), canines (C), premolars (P) and molars (M) are numbered in accordance with their appearance in primitive eutherian dentition (lower-case letter indicates deciduous teeth). [file 13028_2020_521_MOESM1_ESM.pdf]

Identity__________ herd________________ date___________________

Occlusion_________________Supernumerary teeth_______(is drawn) Absence of teeth_______(mark with a ring) Signs of caries(C)_____(mark with *)Fractures(F)____(mark FO/FC, missing part is colored)

Left maxilla								Right maxilla							
		F	C	TW	CI	M	PDI			F	C	TW	CI	M	PDI
i1	I1							i1	I1						
i2	I2							i2	I2						
i3	I3							i3	I3						
c1	C1							c1	C1						
	P1								P1						
p2	P2							p2	P2						
p3	P3							p3	P3						
p4	P4							p4	P4						
	M1								M1						
	M2								M2						
	M3								M3						
Left mandibula								Right mandibula							
i1	I1							i1	I1						
i2	I2							i2	I2						
i3	I3							i3	I3						
c1	C1							c1	C1						
	P1								P1						
p2	P2							p2	P2						
p3	P3							p3	P3						
p4	P4							p4	P4						
	M1								M1						
	M2								M2						
	M3								M3						

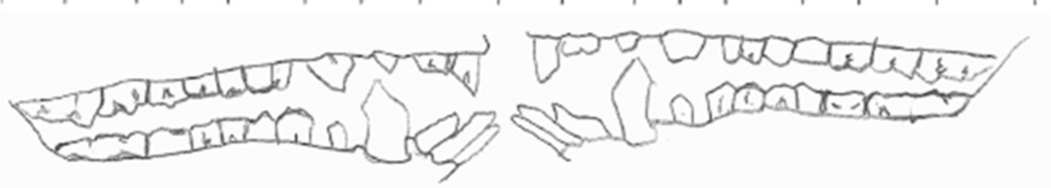

Dental examination chart used in this study. Incisors (I), canines (C), Premolars (P) and molars (M) are numbered in accordance with their appearance in primitive eutherian dentition (lower-case letter indicates deciduous teeth).
