# Supplementary material for: Dental disorders in sows from Swedish commercial herds
Source: Acta Vet Scand. 2020 Jun 4;62:27. doi: 10.1186/s13028-020-00521-7 (PMC7273662; doi:10.1186/s13028-020-00521-7)
Supplement: Supplementary file 2 — Additional file 2: Table S1. Indices used to evaluate dental health in Swedish commercial sows [1] [file 13028_2020_521_MOESM2_ESM.pdf]

Additional file 2

Tabel 1. Indices used to evaluate dental health in Swedish commercial pigs.

Index		Scale	Scale and description
Calculus index (CI)		CI0	No calculi present
		CI1	Calculi covering <50 % of the tooth crown
		CI2	Calculi covering >50 % but not 100 % of the tooth crown
		CI3	Calculi covering 100 % of the tooth crown
Tooth wear (TW)		TW0	No wear
		TW1	Blunting of tooth profile
		TW2	Appearance of reparative dentine
		TW3	Tooth worn to the margin of the gingiva
Mobility (M)		M1	Barely detectable movement
		M2	Tooth is moving but stays in the alveolus
		M3	Very loose tooth that can fall out if touched
Periodontal index (PDI)		PDI1	Gingival recession exposing 20-50 % of root(s)
		PDI2	Gingival recession exposing 50-70 % of root(s), M2, hyperplasia or pus, imminent loss of tooth
		PDI3	Gingival recession exposing >70 % of root(s), M3, appearance of pus and spontaneous tooth loss
